# Environment-Wide Association Study of Blood Pressure in the National Health and Nutrition Examination Survey (1999–2012)

**DOI:** 10.1038/srep30373

**Published:** 2016-07-26

**Authors:** Denise P. McGinnis, John S. Brownstein, Chirag J. Patel

**Affiliations:** 1 Computational Epidemiology, Boston Children’s Hospital, Boston, MA, USA; 2 Department of Biomedical Informatics, Harvard Medical School, Boston, MA, USA

## Abstract

Identifying environmental exposures associated with blood pressure is a priority. Recently, we proposed the environment-wide association study to search for and replicate environmental factors associated with phenotypes. We conducted the environment-wide association study (EWAS) using the National Health and Nutrition Examination Surveys (1999–2012) which evaluated a total of 71,916 participants to prioritize environmental factors associated with systolic and diastolic blood pressure. We searched for factors on participants from survey years 1999–2006 and tentatively replicated findings in participants from years 2007–2012. Finally, we estimated the overall association and performed a second meta-analysis using all survey years (1999–2012). For systolic blood pressure, self-reported alcohol consumption emerged as our top finding (a 0.04 increase in mmHg of systolic blood pressure for 1 standard deviation increase in self-reported alcohol), though the effect size is small. For diastolic blood pressure, urinary cesium was tentatively replicated; however, this factor demonstrated high heterogeneity between populations (I^2^ = 51%). The lack of associations across this wide of an analysis raises the call for a broader search for environmental factors in blood pressure.

Predicting and preventing risk factors for cardiovascular events, such as heart disease and stroke, is a major healthcare concern. One of these risk factors includes hypertension, which is estimated to affect nearly 1 billion adults worldwide and contributes to 7.5 million deaths per year, making it the leading cause of premature death^1^,^2^. The causes of hypertension have been deemed multifactorial, with both hereditary and non-hereditary/environmental factors playing a putative role^3^.

Environmental exposures have been reported as a potential risk for elevations in blood pressure^4^. Blood cadmium^5^,^6^, lead^7^,^8^, polychlorinated biphenyls^9^, dietary nutrients^10^ and particulate matter of size 2.5 microns or smaller^11^,^12^ have all been shown to be associated with moderate to large increases in blood pressure. However, these documented findings are largely confined to studies focused on single pollutants and/or nutrients and may be subject to selection biases and false positive reporting. To address these issues, we have proposed the “environment-wide association study” (or EWAS), a methodology that mimics the analytical steps used in a genome-wide association study (GWAS) to search for new environmental factors in disease and disease-related phenotypes, mitigating the risk for selection biases and false positive reporting. Previously, we have conducted EWAS in type 2 diabetes, preterm birth, all-cause mortality, and other conditions related to heart disease risk, including lipid levels^13^,^14^,^15^,^16^. Furthermore, we utilized a nutrient-wide study approach to search for nutrient factors associated with blood pressure^10^,^17^. In these comprehensive investigations, the entire panel of environmental factors ascertained in the cohort (i.e. folate, enterolactone, self-reported smoking habits, serum cadmium, urinary lead) are evaluated simultaneously in association with a phenotype of interest while controlling for multiple comparisons. Replication is sought for the strongest findings in an independent cohort dataset^13^.

The objective of this investigation is to systematically search 429 environmental factors with respect to blood pressure using the National Health and Nutrition Examination Survey (NHANES) from years 1999–2006 and replicate these associations using NHANES survey data from 2007–2012.

## Results

**Figure 1:**
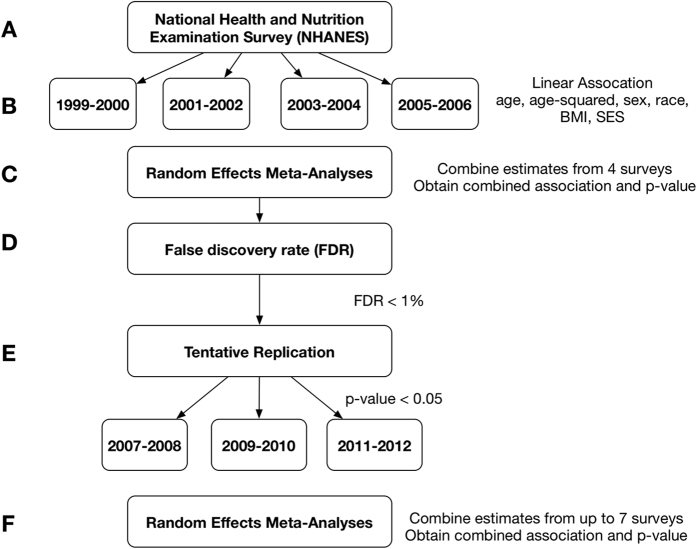
Analysis outline for scan of environmental factors associated with blood pressure. (**A**) NHANES surveys utilized for initial scan. (**B**) Linear regressions to associate each environmental factor with blood pressure (both systolic and diastolic separately). (**C**) Random effects meta-analysis on data from 1999–2006. (**D**) Selected factors with a false discovery rate less than 1%. (**E**) Tentative replication on three surveys from 2007–2012. (**F**) Random effects meta- analysis on combined data from 1999–2012 for tentatively replicated findings.

**Figure 2:**
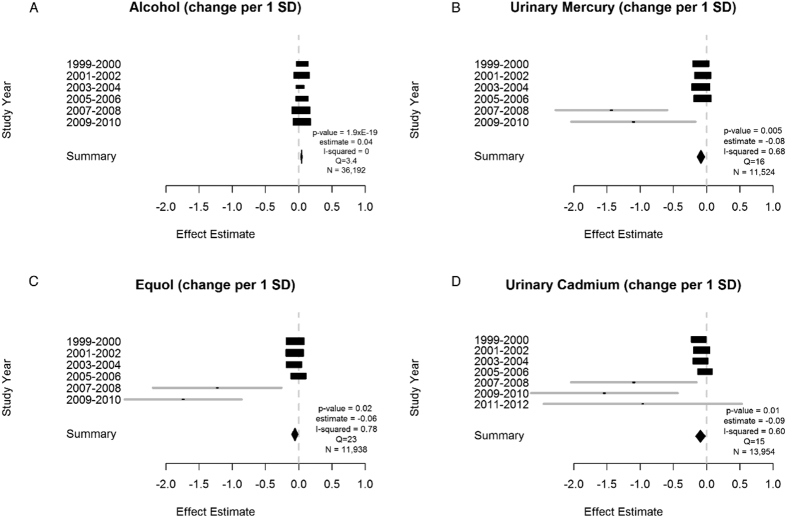
Significant meta-analysis results for systolic blood pressure (1999–2012) represented by forest plots. Additional parameters reported are years of data availability, p-value, effect estimate and total N. I-squared values and Q statistics are included as measures of heterogeneity. N = Number of measured participants.

Environmental factors that achieved an FDR of <1% in the training step are reported for SBP in Table 1. The analytical steps used to scan for these factors are outlined in Fig. 1. Fourteen factors were identified at FDR <1%. Of those, 4 were replicated in one of the 2007–2008, 2009–2010, 2011–2012 surveys shown in Table 2. These 4 replicated factors achieved a significance value of less than 0.05 in the meta-analysis combining all years of data from 1999–2012. The results of the combined analysis are shown in Fig. 2 and represented as forest plots.

Our most robust replicated association was self-reported alcohol consumption (for every 1 standard deviation increase in alcohol there was a 0.04 increase in mmHg of systolic blood pressure, Fig. 2), however this effect size is small. The y-axes of Fig. 2 are comprised of each individual study for which there was available data. The standardized association size is shown for each survey year as a black box (the length of each box represents the 95% confidence interval of each estimate)^18^.

Additionally, urinary mercury, equol (a nonsteroidal estrogen), and urinary cadmium were also significantly replicated all showing inverse associations with SBP with a −0.08, −0.06 and −0.09 decrease in mmHg for every one standard deviation increase, respectively.

**Figure 3:**
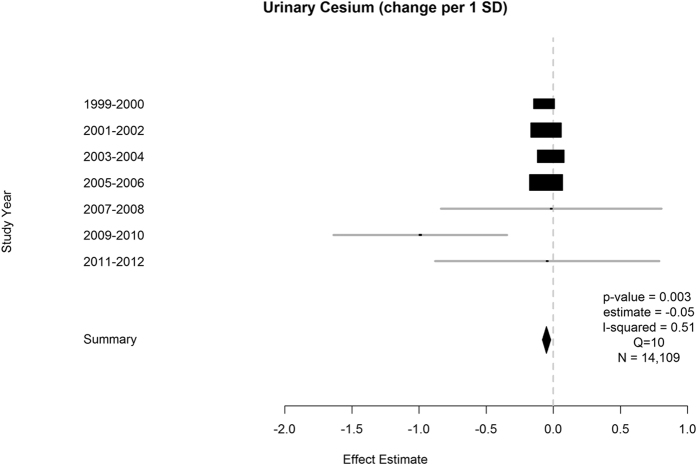
Significant meta-analysis result for diastolic blood pressure (1999–2012) represented by forest plot. Additional parameters reported are years of data availability, p-value, effect estimate and total N. I-squared values and Q statistics are included as measures of heterogeneity. N = Number of measured participants.

Environmental factors that achieved an FDR of <1% in the training step are reported for DBP in Table 3. Urinary cesium was the only factor that was replicated (Table 4 and Fig. 3). A 1 standard deviation increase in logged urinary cesium, corresponded to a 0.05 unit decrease in DBP.

Heterogeneity across surveys for alcohol was negligible (I^2^ = 0). We estimated high heterogeneity for urinary mercury (I^2^ = 68%), equol (I^2^ = 78%) and urinary cadmium (I^2^ = 60%) for SBP and for urinary cesium (I^2^ = 51%) for DBP.

We performed a sensitivity analysis and repeated the regression modeling for the environmental factors that were significant and replicable in Tables 2 and 4 with adjustment for creatinine. Furthermore, we adjusted for serum cotinine, a marker of nicotine to adjust for smoking behavior. These analyses were also performed separately by age (<18 or > = 18) in order to better reflect differences in exposure routes and physiology. After adjustment, the association between alcohol and SBP was significant for the > = 18 age group in the 2009–2010 survey (p = 0.01). Similar results were seen for the association between SBP and equol which was significant for the > = 18 age group in 2009–2010 (p = 0.03). After adjustment, the association between SBP and urinary cadmium for both age groups (<18 and > = 18) was not significant for any survey years. Additionally, the association between urinary cesium and DBP remained significant for the <18 age group in 2009–2010 with a p = 0.04 and an effect estimate nearly 2.5 times larger than the original analysis. Complete results for these sensitivity analyses are reported in Supplementary Tables S1 and S2 for SBP and DBP respectively.

## Conclusions

The purpose of this investigation was to systematically query for cross-sectional associations between environmental factors and blood pressure in a sample representing the general and non-institutionalized population of the US. After consideration of multiple hypotheses and attempting replication, alcohol was the most significant association with systolic blood pressure consistent across all surveys though the effect size was small. This finding has been reported in studies evaluating alcohol and blood pressure including those using NHANES data^19^,^20^,^21^. Excessive alcohol intake has been associated with adverse pathologies such as stroke, cardiomyopathy, cardiodysrhythmia and cirrhosis^22^,^23^.

The remaining factors of urinary mercury, equol and urinary cadmium found in association with SBP must be interpreted with caution. Their measures of heterogeneity are high and dependent on the participants sampled. Nevertheless, Park *et al*. found urinary mercury showed an inverse relationship with SBP in NHANES survey years 2003–2006^24^. The association persisted even after the investigators adjusted for age, gender, race/ethnicity, education, BMI, alcohol, cotinine, omega-3 fatty acids and serum selenium. Park *et al*. attributed this finding to a decrease in kidney function however, mechanisms of mercury toxicity are poorly understood. While animal studies have implied the direction of the association supports this inverse relationship, the dosage amounts cannot be extrapolated to humans^24^,^25^,^26^. In one study, rabbit hearts were isolated and perfused to operate in “working mode” and injected with 2.0 mg/kg of mercuric chloride resulting in the reduction of both systolic and diastolic blood pressures attributed to direct cardiotoxicity^25^. The authors warn these doses far exceed the occupational exposure allowances of humans. For example, the Occupational Health and Safety Administration (OSHA) lists 0.1 mg/m^3^ as the 8-hour time weighted average limit for mercury^25^,^27^. Rossoni *et al*. injected rats with 5 mg/kg of mercuric chloride resulting in decreases to both systolic and diastolic blood pressures^26^. These studies show a link between acute and high-dose exposure and lower blood pressure, but it remains to be seen if chronic low-dose exposure results in cardiovascular disease.

Equol is produced from the metabolism of soy isoflavone by intestinal bacteria and possesses antioxidant properties^28^. *In vitro* studies using “nutritionally relevant plasma concentrations” of equol have found an activation of nitric oxide release and the subsequent relaxation of aortic rings^29^. These effects support a reduction in blood pressure and could explain the inverse associations found in our analysis.

Cadmium, a heavy metal found in tobacco smoke, shellfish, vegetables and ambient air has shown an inconsistent association with blood pressure. Urinary cadmium showed an inverse association with hypertension in a meta-analysis conducted by Gallagher and Meliker^30^ which considered both high and low level exposures^30^. One explanation considered the ability of cadmium to bind to calmodulin increasing levels of dopamine causing a decrease in blood pressure^30^,^31^. However urinary cadmium has also been positively associated with SBP, peripheral artery disease and cardiovascular mortality underlining the inconsistency between study results^32^,^33^.

For diastolic blood pressure, the most significant association was with urinary cesium and lower blood pressure. Cesium exposure is most likely to occur through diet but can also occur through inhalation. In general, cesium levels in the environment are very low and unlikely to pose a significant health threat^34^. Cesium chloride has been promoted as a homeopathic cancer therapy where excessive ingestion of stable cesium has been associated with cardiac arrhythmias^34^. It is likely the relationship found in our study is explained by unmeasured confounding not accounted for in the model.

This analysis was not without limitations. The purpose of this analysis was to screen for environmental factors to guide more in-depth research of exposure-related health effects, therefore regression models included both adults and children over age 8. Despite controlling for age within the regression model, careful consideration should be exercised when evaluating causality as physiological differences exist between adults and children with respect to exposure routes, patterns and susceptibilities^35^.

Another important limitation is the uncertainty introduced into statistical models in measurement of biological specimens. Concentrations of the target compound in proxy tissue reflect what was present at the time of collection contingent on fasting and hydration protocols adhered to prior to testing^36^. Therefore, there is an increased chance of both false positives and negatives. Lack of adjustment for this phenomenon might explain the inverse association between urinary cadmium/cesium and blood pressure in this analysis. However, consensus on the proper adjustment for fasting and hydration has not been established. One approach has been to include urinary creatinine levels in regression models to account for variability in urine diluteness^36^,^37^. To address the issue of this bias, we performed a sensitivity analysis as described previously and found that even after adjustment for cotinine and urinary creatinine, the association between alcohol and blood pressure is still nominally significant in 2009–2010 (p = 0.01). Equol is also nominally significant after adjustment in adults in the 2009–2010 survey (p = 0.03). Cesium and cadmium are not significant in models adjusted for serum cotinine and urinary creatinine in adults.

While we combined all years of data, some meta-analyses had low power to detect significant associations. The possibility of a U-shaped association with alcohol may also not have been captured by the linear models utilized, where blood pressure is higher in non/high drinkers and lowest in moderate drinkers^38^. Furthermore, while we scanned up to 429 environmental factors we only had the opportunity to replicate 140 as we did not have complete measures for all years. Due to changes in measurement techniques across years (i.e. serum folate replaced with red blood cell folate), missing data may have contributed to the lack of replicated findings. We attempted to adjust for major contributors of confounding and chose covariates connected with both blood pressure and numerous exposures, such as socio-economic status^39^. NHANES is cross-sectional; therefore, these results suggest associations contributing to disease and findings may be reverse-causal (e.g., exposure coming after changes in blood pressure). Such a study cannot replace a prospective study to mitigate chances for reverse causality.

Despite these limitations, we were able to comprehensively analyze up to 13 years of the NHANES survey, consisting of ~71,000 individuals. Because of the relatively few replicated findings and evidence for sizable association heterogeneity, we believe that there is a need to go beyond the 429 queried here to discover factors that describe variation in phenotype in blood pressure that is not explained by hereditary factors. For example, recently investigators have queried 7100 factors related to the metabolome in association with blood pressure^40^. As a comprehensive array of the products of metabolism of environmental exposure and diet (as well as endogenous processes), the metabolome shows promise to capture a large fraction of the human exposome^41^.

Aside from alcohol, we identified factors that possessed such high levels of heterogeneity their associations are weak at best. We believe it is important to report this negative outcome as some studies that select specific exposures and/or outcomes could be guilty of reporting bias where enough stratification will often produce a positive result. The strength of the association between alcohol and SBP confirms an already large body of knowledge that usage of alcohol affects blood pressure^42^,^43^.

We believe the current analysis was the first of its kind to systematically evaluate this number of environmental exposures across this large of a time frame in a large sample representative of the United States. We have recapitulated an association between alcohol and blood pressure. Furthermore, we report all associations queried to avoid misinterpretation that may result when a variety of data manipulations (e.g., stratifications, transformations) and analytic techniques (e.g., modeling techniques) are employed, particularly in large and accessible datasets like NHANES^44^,^45^. The lack of associations across this wide of an analysis raises the call for a broader search for environmental factors in blood pressure.

## Methods

### Study Population

Data for this analysis was attained from NHANES, a nationally representative sample of the US civilian, non-institutionalized population conducted by the US Centers for Disease Control and Prevention (CDC) every two years^46^. This cross-sectional dataset is comprised of health questionnaire, laboratory (i.e. urinary phthalates, blood lead, blood cadmium, urinary mercury), and clinical data using a multistage probability sampling design^47^,^48^. Data was collected through in-person interviews, physical measurement at mobile examination centers and laboratory samples. Protocol approval and written informed consent was obtained by the National Center for Health Statistics Institutional Review Board for participants >18 years of age and from the guardians of participants <18. All methods were carried out in accordance with the approved guidelines. All survey and consent documents for NHANES were approved by the CDC Institutional Review Board. This study was conducted in accordance with the STROBE guidelines (Strengthening the Reporting of Observational Studies in Epidemiology)^49^.

The entire dataset was comprised of the 1999–2000, 2001–2002, 2003–2004, 2005–2006, 2007–2008, 2009–2010, and 2011–2012 surveys which evaluated 9965, 11039, 10122, 10348, 10149, 10537 and 9756 participants respectively. However, environmental factors were assessed in different subsets of the population and the median sample sizes for the 1999–2000, 2001–2002, 2003–2004, 2005–2006, 2007–2008, 2009–2010, and 2011–2012 surveys was 1607, 2097, 1905, 5825, 2170, 2360, and 1973, respectively.

### Blood Pressure Measures

Blood pressure is measured in all study participants that are 8 years or older using a mercury manometer. Participants are asked to be seated for 5 minutes after which three consecutive blood pressure measurements are taken on the same arm with a 30 second wait in between measures. If any of the previous three measures was interrupted or one or more of the readings could not be made, a fourth measurement was made. The mean of the systolic and diastolic measurements was used as the dependent variable in these analyses^50^,^51^,^52^,^53^.

### Statistical Analysis

Figure 1 shows the analysis steps used to scan for factors associated with either systolic (SBP) or diastolic (DBP) blood pressure. A total of 429 factors were evaluated for 1999–2000, 2001–2002, 2003–2004, and 2005–2006 (Fig. 1A). Each year a varying number of factors were measured, 1999–2000 (N = 379), 2001–2002 (N = 399), 2003–2004 (N = 354), 2005–2006 (N = 256), however a False Discovery Rate (FDR) was able to be calculated for 429 environmental factors overall. A series of survey-weighted linear regressions controlling for age, age^2^, sex, race, body mass index (BMI) and socioeconomic status (SES) are performed on surveys from 1999–2006 to establish a training set of significant associations (Fig. 1B). Race/ethnicity was categorized according to the methodology of Patel *et al*.^15^ and included Non-Hispanic White, Mexican American, Non-Hispanic Black, Other Hispanic and Other^15^. Body mass index was calculated using measured values for height and weight for study participants: weight(kilograms)/height(meters)^54^. Socioeconomic status was estimated by dividing the participant’s household income by the time-adjusted poverty threshold as described in Patel *et al*.^13^. We chose these covariates based on their association with blood pressure^55^,^56^,^57^.

Continuous factors with skewed distributions were log-transformed and z-standardized in order to compare association sizes across all factors. To increase power for discovery, we performed a random-effects meta-analysis to combine associations from the 1999–2000, 2001–2002, 2003–2004 and 2005–2006 surveys respectively (Fig. 1C)^58^. We estimated the false discovery rate (FDR) to control for the proportion of significant results that are false positives due to chance^59^. Of those deemed significant at an FDR threshold less than 1% in the meta-analysis of years 1999–2006 (Fig. 1D) and had measurements in 2007–2012 (N = 140), we sought independent replication in any of the 2007–2008, 2009–2010, or 2011–2012 surveys with a nominal significance level of p < 0.05 (Fig. 1E).

Finally, we report an overall meta-analysis integrating data from all surveys 1999–2012 with factors that achieved an FDR < 1% in the training set and were tentatively replicated in the testing datasets (Fig. 1F). These meta-analyses are graphically represented using forest plots for SBP and DBP (Figs 2 and 3), along with measures of heterogeneity (I^2^ and Q) and the total number of measurements. The Q statistic indicates heterogeneity and the I^2^ value is a measure of the percentage of variation across studies that is due to heterogeneity and not chance^58^,^60^. For all analyses, the R survey and *rmeta* libraries were utilized to account for survey weights, strata and complex sampling structure^15^,^61^,^62^.

## Additional Information

**How to cite this article**: McGinnis, D. P. *et al*. Environment-Wide Association Study of Blood Pressure in the National Health and Nutrition Examination Survey (1999–2012). *Sci. Rep.* **6**, 30373; doi: 10.1038/srep30373 (2016).

## Supplementary Material

Supplementary Information

## Figures and Tables

**Table 1:** Meta-analysis results from survey years 1999–2006 with a threshold of FDR < 1% for systolic blood pressure (SBP).

SBP	Random Effects Meta-Analysis 1999–2006 (training dataset)			
Variable Name (change per 1 SD of the logarithm of factor)	Effect Size (mmHg)	p-value	Total N	FDR
Alcohol	0.04	1.4 × 10^−11^	22986	2.0 × 10^−9^
Enterolactone	−0.08	2.0 × 10^−11^	7850	2.2 × 10^−9^
Equol	−0.05	1.9 × 10^−10^	7597	1.3 × 10^−8^
Mercury, urine	−0.08	2.2 × 10^−8^	6986	1.3 × 10^−6^
Mono-(3-carboxypropyl) phthalate	−0.05	3.1 × 10^−6^	6081	0.0001
Folate, serum	−0.05	4.1 × 10^−6^	22269	0.0002
2,5-dichlorophenol	−0.06	4.4 × 10^−6^	7547	0.0002
Cadmium, urine	−0.08	1.1 × 10^−5^	7457	0.0003
4-fluoro-3-phenoxybenzoic acid	−0.02	1.5 × 10^−5^	3647	0.0004
Vitamin A	−0.03	7.0 × 10^−5^	17829	0.002
Retinol	−0.03	9.0 × 10^−5^	17829	0.002
Thallium, urine	−0.07	0.0002	7549	0.003
Cesium, urine	−0.07	0.0003	7613	0.004
Cadmium	0.03	0.0003	22493	0.005

Variables were log transformed and z-standardized therefore results reflect a change per 1 standard deviation (SD). FDR = False Discovery Rate. N = number of participants measured.

**Table 2:** List of environmental factors with an FDR < 1% in 1999–2006 meta-analysis that replicated in at least one survey between 2007–2012 with a p < 0.05 for systolic blood pressure.

SBP	2007–2008			2009–2010			2011–2012		
Variable Name (change per 1 SD of the logarithm of factor)	Effect Size (mmHg)	95% CI	p-value	Effect Size (mmHg)	95% CI	p-value	Effect Size (mmHg)	95% CI	p-value
Alcohol	0.03	[0.02, 0.05]	0.011	0.05	[0.03, 0.06]	0.003	NA	NA	NA
Mercury, urine	−1.43	[−2.27, −0.06]	0.016	−1.10	[−2.04, −0.17]	0.060	NA	NA	NA
Equol	−1.23	[−2.20, −0.26]	0.050	−1.74	[−2.62, −0.86]	0.008	NA	NA	NA
Cadmium, urine	−1.10	[−2.04, −0.02]	0.063	−1.54	[−2.64, −0.43]	0.034	−0.96	[−2.46, 0.53]	0.247

SD = Standard Deviation. CI = Confidence Interval. NA = Not Available.

**Table 3:** Meta-analysis results from survey years 1999–2006 with a threshold of FDR < 1% for diastolic blood pressure (DBP).

DBP	Random Effects Meta-Analysis 1999–2006 (training dataset)			
Variable Name (change per 1 SD of the logarithm of factor)	Effect Size (mmHg)	p-value	Total N	FDR
Cesium, urine	−0.05	1.3 × 10^−7^	7613	1.5 × 10^−5^
# Cigs smoked/day now	−0.06	1.6 × 10^−6^	5202	0.0001
1,2,3,4,7,8-hexachlorodibenzo-p-dioxin	0.09	7.8 × 10^−6^	2293	0.001
Dodecanoic Acid	0.02	5.8 × 10^−5^	22986	0.003
# Cigs/day past 30 days	−0.07	7.8 × 10^−5^	4357	0.004
3,3,4,4,5,5-hexachlorobiphenyl	0.11	8.6 × 10^−5^	3640	0.004

Variables were log transformed and z-standardized therefore results reflect a change per 1 standard deviation (SD). SD = Standard Deviation. FDR = False Discovery Rate. N = number of participants measured.

**Table 4:** List of environmental factors with an FDR < 1% in 1999–2006 meta-analysis that replicated in at least one survey between 2007–2012 with a p < 0.05 for diastolic blood pressure.

DBP	2007–2008			2009–2010			2011–2012		
Variable Name (change per 1 SD of the logarithm of factor)	Effect Size (mmHg)	95% CI	p-value	Effect Size (mmHg)	95% CI	p-value	Effect Size (mmHg)	95% CI	p-value
Cesium, urine	−0.02	[−0.84,0.81]	0.972	−0.99	[−1.64,−0.35]	0.024	−0.05	[−0.88,−0.79]	0.917

SD = Standard Deviation. CI = Confidence Interval. NA = Not Available.
