# Supplementary material for: Environment-Wide Association Study of Blood Pressure in the National Health and Nutrition Examination Survey (1999–2012)
Source: Sci Rep. 2016 Jul 26;6:30373. doi: 10.1038/srep30373 (PMC4960597; doi:10.1038/srep30373)
Supplement: Supplementary Information [file srep30373-s1.pdf]

Environment-Wide Association Study of Blood Pressure in the National Health and Nutrition Examination Survey (1999-2012)

Denise P. McGinnis[1], John S. Brownstein[1], *Chirag J. Patel[2]

A)

SBP (all ages)	2007-2008			2009-2010			2011-2012		
**Variable Name (change per 1 SD of the logarithm of factor)**	**Effect Size (mmHg)**	**95% CI**	**p-value**	**Effect Size (mmHg)**	**95%CI**	**p-value**	**Effect Size (mmHg)**	**95%CI**	**p-value**
Alcohol	0.03	[0.02,0.05]	0.011	0.05	[0.03,0.06]	0.003	NA	NA	NA
Mercury, urine	-1.43	[-2.27,0.06]	0.016	-1.10	[-2.04,-0.17]	0.060	NA	NA	NA
Equol	-1.23	[-2.20,0.26]	0.050	-1.74	[-2.62,-0.86]	0.008	NA	NA	NA
Cadmium, urine	-1.10	[-2.04,0.02]	0.063	-1.54	[-2.64,-0.43]	0.034	-0.96	[-2.46,0.53]	0.247

B)

SBP (age<18 years)	2007-2008			2009-2010			2011-2012		
**Variable Name (change per 1 SD of the logarithm of factor)**	**Effect Size (mmHg)**	**95% CI**	**p-value**	**Effect Size (mmHg)**	**95%CI**	**p-value**	**Effect Size (mmHg)**	**95%CI**	**p-value**
Alcohol	-0.01	[-0.11,0.09]	0.816	-0.12	[0.31,0.78]	0.302	NA	NA	NA
Mercury, urine	-0.76	[-2.30,0.78]	0.387	0.14	[-0.86,1.14]	0.801	NA	NA	NA
Equol	0.17	[0.85,1.20]	0.760	-0.82	[-1.55,-0.09]	0.094	NA	NA	NA
Cadmium, urine	0.09	[-0.56,0.75]	0.793	-0.91	[-2.35,0.54]	0.285	-1.10	[-2.26,0.06]	0.120

SBP (age>=18 years)	2007-2008			2009-2010			2011-2012		
**Variable Name (change per 1 SD of the logarithm of factor)**	**Effect Size (mmHg)**	**95% CI**	**p-value**	**Effect Size (mmHg)**	**95%CI**	**p-value**	**Effect Size (mmHg)**	**95%CI**	**p-value**
Alcohol	0.03	[0.01,0.05]	0.071	0.05	[0.03,0.07]	0.010	NA	NA	NA
Mercury, urine	-1.36	[-2.47,0.25]	0.075	-0.48	[-1.57,0.60]	0.433	NA	NA	NA
Equol	-1.71	[-3.15,0.27]	0.081	-1.58	[-2.52,-0.64]	0.030	NA	NA	NA
Cadmium, urine	-0.91	[-2.93,1.11]	0.428	-0.16	[-1.83,1.50]	0.856	-0.66	[2.34,1.03]	0.480

Supplemental Table S1. List of environmental factors with an FDR<1% in 1999-2006 meta-analysis that replicated in at least one survey between 2007-2012 with a p<.05 for systolic blood pressure. A) Results for original model adjusted for age, sex, BMI, age-squared, poverty and race/ethnicity for all ages combined. B) Results after additional adjustment for creatinine and cotinine for ages<18 years .C) Results after additional adjustment for creatinine and cotinine for ages>=18 years. SD=Standard Deviation. CI=Confidence Interval. NA=Not Available.

A)

DBP (all ages)	2007-2008			2009-2010			2011-2012		
**Variable Name (change per 1 SD of the logarithm of factor)**	**Effect Size (mmHg)**	**95% CI**	**p-value**	**Effect Size (mmHg)**	**95% CI**	**p-value**	**Effect Size (mmHg)**	**95% CI**	**p-value**
Cesium, urine	-0.02	[-0.84,0.81]	0.972	-0.99	[-1.64,-0.35]	0.024	-0.05	[-0.88, 0.79]	0.917

B)

DBP (age<18 years)	2007-2008			2009-2010			2011-2012		
**Variable Name (change per 1 SD of the logarithm of factor)**	**Effect Size (mmHg)**	**95% CI**	**p-value**	**Effect Size (mmHg)**	**95% CI**	**p-value**	**Effect Size (mmHg)**	**95% CI**	**p-value**
Cesium, urine	0.52	[-1.19, 2.23]	0.582	-3.45	[-5.71, -1.18]	0.041	2.81	[-0.70,6.32]	0.178

C)

DBP (age>=18 years)	2007-2008			2009-2010			2011-2012		
**Variable Name (change per 1 SD of the logarithm of factor)**	**Effect Size (mmHg)**	**95% CI**	**p-value**	**Effect Size (mmHg)**	**95% CI**	**p-value**	**Effect Size (mmHg)**	**95% CI**	**p-value**
Cesium, urine	-0.005	[-1.42, 1.41]	0.995	-0.69	[-1.97, 0.59]	0.350	0.18	[-1.00,1.35]	0.782

Supplemental Table S2. List of environmental factors with an FDR<1% in 1999-2006 meta-analysis that replicated in at least one survey between 2007-2012 with a p<.05 for diastolic blood pressure. A) Results for original model adjusted for age, sex, BMI, age-squared, poverty and race/ethnicity for all ages combined. B) Results after additional adjustment for creatinine and cotinine for ages<18 years. C) Results after additional adjustment for creatinine and cotinine for ages>=18 years. SD=Standard Deviation. CI=Confidence Interval. NA=Not Available
